# Aldosterone-identified targets for optimal sodium and potassium supplementation in intestinal failure

**DOI:** 10.3389/fnut.2026.1864539

**Published:** 2026-07-14

**Authors:** Robert H. Foerster, Georg Lamprecht, Mads V. Sørensen, Jens Leipziger, Peder Berg

**Affiliations:** 1 Division of Gastroenterology, Hepatology and Nutritional Medicine, Department of Internal Medicine, Rostock University Medical Center, Rostock, Germany; 2 Department of Biomedicine, Health, Aarhus University, Aarhus, Denmark

**Keywords:** aldosterone, intestinal failure, parenteral support, potassium homeostasis, short bowel, sodium homeostasis

## Abstract

**Background:**

Parenteral support (PS) is a life-saving organ replacement therapy for patients with intestinal failure (IF). However, practical strategies to establish optimal PS sodium and potassium remain poorly defined. We investigated whether elevated plasma renin and aldosterone reflect suboptimal electrolyte supplementation and could help guide physiologically optimal sodium and potassium supplementation in patients with IF.

**Methods:**

Real-world monitoring data from adult IF patients receiving long-term PS were analyzed using mixed-effects models for repeated measurements. Associations between parenteral sodium and potassium supplementation, urinary sodium and potassium excretion, and plasma renin and aldosterone were studied across short bowel patients with a jejunostomy (SB-J) or with colon in continuity (SB-CiC).

**Results:**

In 618 visits from 110 patients, elevated aldosterone levels occurred frequently and were more common in SB-J (adjusted OR for hyperaldosteronism: 5.2; 95% CI 1.5 to 17.8; *p* = 0.009). Even after adjusting for sodium and volume depletion, SB-J had significantly higher aldosterone levels (2.1-fold higher; 95% CI 1.5 to 2.9; *p* < 0.001). Urinary potassium excretion increased with greater potassium infusion and was significantly higher in SB-J (adjusted difference: 28 mmol/d; 95% CI 9 to 47; *p* < 0.001). A urinary sodium >20 mmol/L, combined with a sodium-to-potassium ratio >1, ruled out hyperaldosteronism with a negative predictive value of 93%.

**Conclusion:**

Aldosterone not only reflects sodium depletion but is also stimulated to excrete overzealous intravenous potassium supplementation in patients with IF. Targeting a urinary sodium concentration of >20 mmol/L and a urinary sodium-to-potassium ratio of >1 can help to guide physiologically optimal sodium and potassium support in IF. Subsequently, parenteral potassium support of less than 1.0 mmol/kg/d is likely sufficient for most patients with IF.

## 1 Introduction

Intestinal failure (IF) is defined by the need for parenteral support (PS) of water, electrolytes, and, in many cases, macronutrients to sustain life and growth (1). PS for IF is therefore an organ-replacement therapy comparable to dialysis for end-stage renal failure. IF is most often caused by short bowel (SB) as a result of surgical resection involving the small intestine (2). Postoperative functional anatomy is categorized as end-jejunostomy (SB-J), jejunocolonic anastomosis (SB-JC), or jejunoileocolonic anastomosis (SB-JIC). The latter two are often taken together as short bowel with colon in continuity (SB-CiC) (3).

Stoma fluid losses in patients with SB-J are usually greater than 2 L, typically exceeding diarrheal losses in patients with SB-CiC (4). In addition, it is thought that short bowel patients with end-jejunostomy have generally higher intestinal electrolyte losses than patients with SB-CiC.

Individual composition of PS, so-called compounding, is often necessary, but current guidelines do not provide operationalized recommendations for optimal electrolyte supplementation (5). Monitoring of 24-h urine output to guide the volume of PS is clinically well established. It has been used in recent pivotal trials evaluating the effect of Glucagon-like peptide-2 (GLP-2) analogues to increase intestinal absorption (6, 7).

In contrast, there is no clear consensus on how sodium and potassium support should be guided. The conventional approach is to replenish estimated intestinal sodium loss based on 100 mmol sodium per liter of small bowel stoma output and 30 mmol sodium per liter of colonic stool (8, 9). Parenteral potassium is typically administered according to generalized weight-based recommendations from the recent European guideline (1.0–1.5 mmol/kg/d) (5).

Building on the approach of optimal volume (water) support, we aimed to operationalize parenteral sodium and potassium support in IF more rigorously based on physiological regulatory mechanisms. The renin-angiotensin-aldosterone system (RAAS) acts to rebalance volume and sodium homeostasis by increasing the absorption of sodium and, consequently, water in the kidneys (10). RAAS is stimulated by low sodium delivery to the macula densa, low renal plasma flow, and sympathetic activity (11). Therefore, hyperaldosteronism is regarded as a biomarker of volume and sodium depletion (12). We reasoned that optimal sodium and volume support should be defined by a relaxed renin-angiotensin-aldosterone axis, i.e., aldosterone levels within the normal range.

In addition to its role in sodium reabsorption in the distal renal tubular system, aldosterone is a potent stimulator of renal and colonic potassium excretion. Small increases in extracellular potassium trigger voltage-induced calcium influx into adrenal glomerulosa cells, directly stimulating the acute and chronic release of aldosterone (13–15).

Given their role in regulating volume, sodium, and potassium homeostasis, we analyzed renin and aldosterone to either indicate optimal or to detect suboptimal supplementation of parenteral sodium and potassium in patients with short bowel and intestinal failure. To do this, we used mixed-effects modeling to establish physiologically optimal parenteral supplementation of sodium and potassium in intestinal failure.

## 2 Material and methods

### 2.1 Sex as a biological variable

There was a similar representation of males and females in the study cohort. Where relevant, the results from sex-stratified analyses are given.

### 2.2 Patient cohort

**Figure 1:**
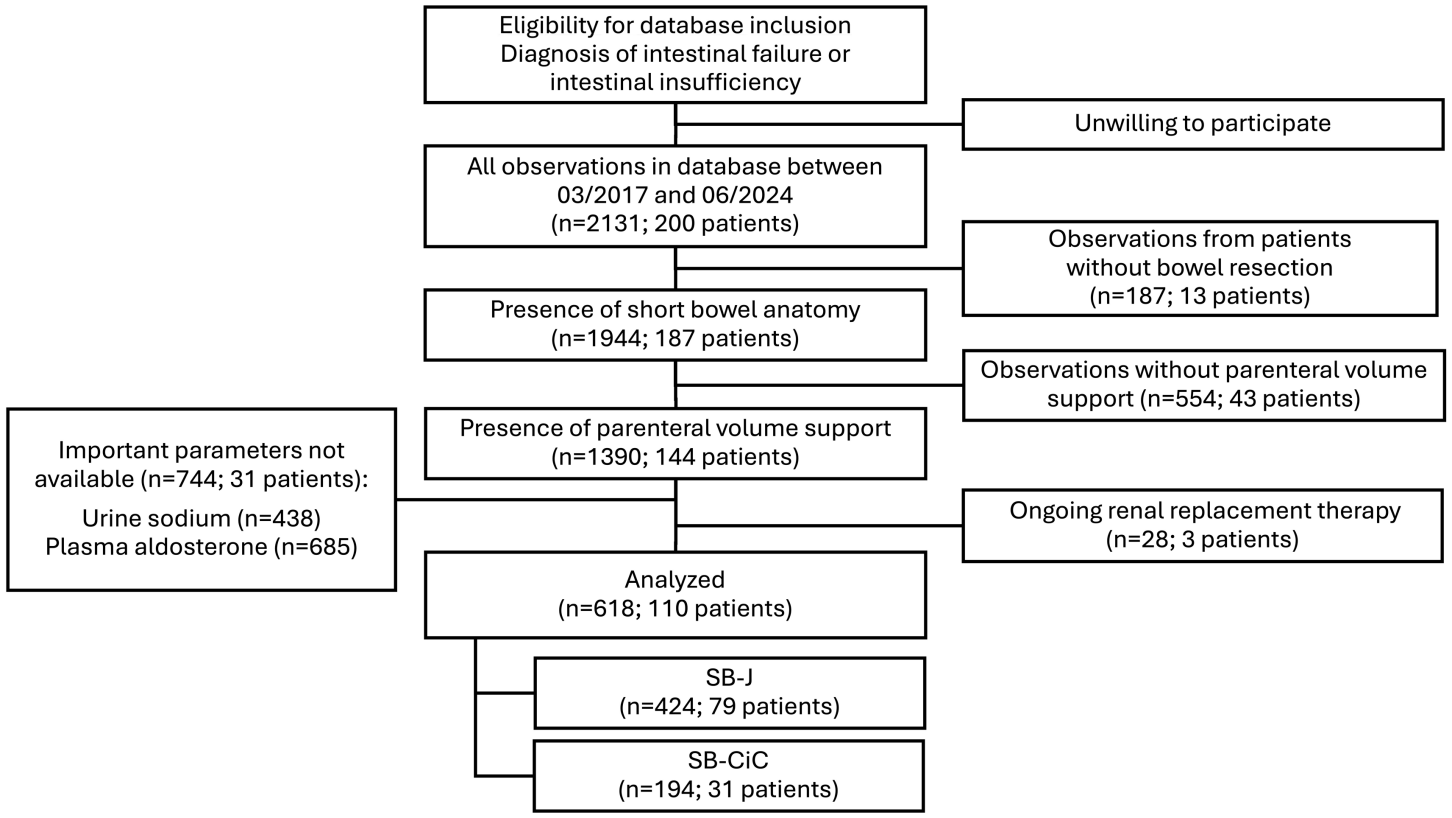
Flow diagram of data inclusion.

All visits of patients older than 18 years with chronic intestinal failure or intestinal insufficiency who presented in the outpatient clinic of the University Medical Center Rostock and provided written informed consent were included into our prospectively customized database (Microsoft Access, 2010). The study (German Clinical Trials Register: DRKS00021085) was approved by the local ethics committee according to German law (A 2018–0128). A diagram of data inclusion is shown in Figure 1. Patient visits were included in this analysis if the following criteria were fulfilled: presence of short bowel anatomy (SB-J: end-jejunostomy, SB-JC: jejunocolonic anastomosis with colon in continuity, SB-JIC: jejunoileocolonic anastomosis with colon in continuity); presence of parenteral volume support (>0 L/week); availability of serum aldosterone, and urine sodium measurements. SB-JC and SB-JIC were grouped into one category termed SB-CiC (short bowel with colon in continuity). We included visits between March 2017 and June 2024.

### 2.3 Local standards of adjusting parenteral support

All patients received treatment in accordance with recent ESPEN guidelines (5, 16). Patients on long-term parenteral support had regularly scheduled follow-up visits every 3–6 months. Serum electrolytes and 24h urine measurements were part of the clinical routine and influenced clinical decisions. Serum hormones (aldosterone and renin) were exploratorily measured within the routine outpatient setting. Decisions on PS adjustments were not regularly based on result reports for serum hormones. Based on pathophysiological considerations and local clinical experience, urinary sodium/potassium ratio <1 has been regarded as a sign of latent hypovolemia (10). Low urinary sodium excretion (<40 mmol/d or <20 mmol/L) indicated a need for more sodium support. Patients were not restricted in their intake of oral food and beverages.

### 2.4 Data handling

We recorded the composition of PS from pharmacy prescriptions of the visit prior to laboratory sampling. PS volume, macronutrients (amino acid, glucose, lipid), and electrolytes (sodium, potassium, calcium, magnesium, phosphate, acetate, chloride) from standard or compounded nutrition bags and additional fluid infusions were captured. All components were added up per week and divided by 7 days. Information on remaining small bowel length was preferentially taken from the surgical reports, or the length of the resected bowel was subtracted from the presumed initial length of 350 cm (17). Colon length was included as percentage remaining colon according to Cummings et al. (18). Missing values were imputed using intraindividual linear regression for renin (10, 1.6%), creatinine (12, 1.9%), serum potassium (12, 1.9%), and sodium (8, 1.3%). Missing values of urine volume (163, 26.4%) and urinary potassium (4, 0.6%) were not imputed.

### 2.5 Urine and blood analytics

Routine blood and urine tests were performed by the central laboratory of the University Medical Center Rostock. Blood samples were taken in the morning, while sitting, and without specified resting time. Blood parameters included serum electrolytes, venous blood gas analysis, creatinine, renin, and aldosterone. Measurements of renin (radioimmunoassay, ACTIVE® Renin IRMA, Beckman Coulter, US) and plasma aldosterone (LC–MS/MS, MassChrom® Steroide, Chromsystems Instruments, Germany) were performed by using commercially available assays. Estimated glomerular filtration rate (eGFR) was calculated with Chronic Kidney Disease Epidemiology Collaboration (CKD-EPI) equation (2009) based on serum creatinine (19). Urine electrolytes were measured in 24h urine collection samples or spot urine samples. Urine volumes were measured and reported by the patients similar to the procedures in the STEPS and EASE trials (6, 7).

### 2.6 Control cohort

Plasma aldosterone and renin concentrations were obtained from a German regional cohort of 1347 healthy subjects. Radioimmunometric procedures were used to measure both aldosterone (Coat-A-Count Aldosterone, Siemens Healthcare Diagnostics, Eschborn, Germany) and renin (Renin III generation, Cisbio Bioassay, Bagnols-sur-Cèze Cedex, France) (20).

### 2.7 Statistics

We used R (version 4.4.2, Mac) for data transformation. Data analysis and graphical depiction of results were done using Stata 17 for Mac (StataCorp). Data distribution was assessed by quantile-quantile plots. To include all available data while accounting for multiple samples per patient, linear mixed effects models for repeated measures were used to estimate differences between SB types and for association analyses. Patient characteristics and PS details were assessed by summary statistics. Odds ratios were calculated using logistic mixed-effects models for repeated measures. All tests were two-sided and performed at a total significance level of 5%. Specifics for statistical testing are provided with each table and figure. Error bars represent the standard error of the mean unless otherwise specified.

#### 2.7.1 Linear mixed-effect model

The main outcome of interest was the plasma aldosterone level. Plasma aldosterone was log-transformed before statistical analysis, and results were back-transformed to the original scale to allow easier interpretation. As plasma aldosterone followed a log-normal distribution, the back-transformed estimates (the geometric mean) correspond closely to the median.

To analyze aldosterone levels with respect to multiple associations, we used a mixed-effect regression model for repeated measures to adjust for the differences in multiple parameters. By default, the full model included anatomy type (SB-J vs. CIC), eGFR, plasma potassium, plasma sodium, urine volume, and urinary sodium excretion as fixed effect variables (all continuous except anatomy type) and patient pseudonym as random effect variable. These full models only include observations for which 24h urine collections were available since the models integrated urine volume and 24h electrolyte excretion. The parameters were estimated using restricted maximum likelihood, expressing medians with standard error intervals. Only if the model estimated a non-linear correlation that was significantly different from linear, we rejected the assumption of a linear correlation for these parameters. Following this, all fixed effect variables except plasma potassium (linear modeling) and anatomy type were modeled as restricted cubic splines. If allowing an interaction between anatomy type and the parameter resulted in significantly different slopes for each anatomy type, we included this interaction term in the model. Otherwise, the slopes were assumed to be identical, independent of anatomy type. For all variables except anatomy type and the predictor of interest, prediction of associations was performed at the mean of the overall cohort. Sensitivity analyses were performed on subgroups that excluded patients with an eGFR <30 mL/min/1.73m^2^ or with any imputed data (Supplementary Tables 2–10).

## 3 Results

### 3.1 Characteristics of the cohort

We included data from 618 outpatient visits involving 110 patients (76% of all patients in our database who received parenteral support during the study period). During the observation period, 18 patients underwent bowel surgery that changed their SB anatomical type (reconstruction SB-J to SB-JC (*n* = 12) or reconstruction of SB-J to SB-JIC (*n* = 5), new jejunostomy SB-JIC to SB-J (*n* = 1)). All patients on dialysis (*n* = 3) were excluded *a priori*.

Demographic, anatomical, and clinical characteristics are shown in Table 1. SB-CiC included 147 observations from patients with SB-JC and 47 observations from patients with SB-JIC. The relevant concomitant medication is reported in Supplementary Table 1.

**Table 1:** Patient characteristics.

Parameter	Total	SB-J	SB-CiC
At first visit			
Number of patients, *n*	110	79	31
Age in years, mean (SD)	56.9 (14.8)	55.6 (15.4)	60.2 (12.8)
Female, *n* (%)	59 (54)	41 (52)	18 (58)
Colon length in %, mean (SD)			68 (23)
Small bowel length in cm, mean (SD)	162 (98)	164 (91)	155 (116)
BMI in kg/m^2^, mean (SD)	23.3 (5.7)	23.7 (6.0)	22.3 (4.6)
Serum albumin in g/L, mean (SD)	37.8 (5.1)	38.4 (5.3)	35.9 (4.1)
eGFR in mL/min/1.73 m^2^, mean (SD)	76.7 (30.8)	76.1 (32.2)	78.2 (27.3)
eGFR <30 mL/min/1.73 m^2^, *n* of patients (%)	5 (5)	3 (4)	2 (6)
Urine volume in L/d, mean (SD)	1.6 (0.7)	1.6 (0.7)	1.7 (0.7)
Urine creatinine in mmol/d, mean (SD)	9.7 (4.9)	9.8 (4.1)	9.6 (6.7)
PS-Volume in mL/kg/d, mean (SD)	33.8 (17.0)	36.4 (18.2)	27.1 (11.0)
PS-Energy in kcal/kg/d, mean (SD)	16.5 (9.2)	16.5 (9.6)	16.5 (8.1)
All observations			
Number of observations, *n*	618	424	194
Observations per patient, median (min-max)	3 (1–26)	3 (1–26)	2 (1–18)
Observation period in months, median (min-max)	12 (0–84)	14 (0–84)	8 (0–84)
eGFR <30 mL/min/1.73 m^2^, *n* of observations (%)	27 (4)	14 (3)	13 (7)
24h urine samples available, *n* (%)	455 (74)	316 (75)	139 (72)

Patient characteristics were recorded at the first visit and number of observations per patient. During the observation period, no values were available for serum albumin in three patients (2.7%), urine volume in 16 patients (14.5%), and urine creatinine excretion in 30 patients (27.3%). SB-J, short bowel with end-jejunostomy; SB-CiC, short bowel with colon in continuity; eGFR, estimated glomerular filtration rate.

### 3.2 Parenteral volume, sodium, and potassium support as well as the corresponding urinary excretion differ significantly between anatomical types of SB

**Figure 2:**
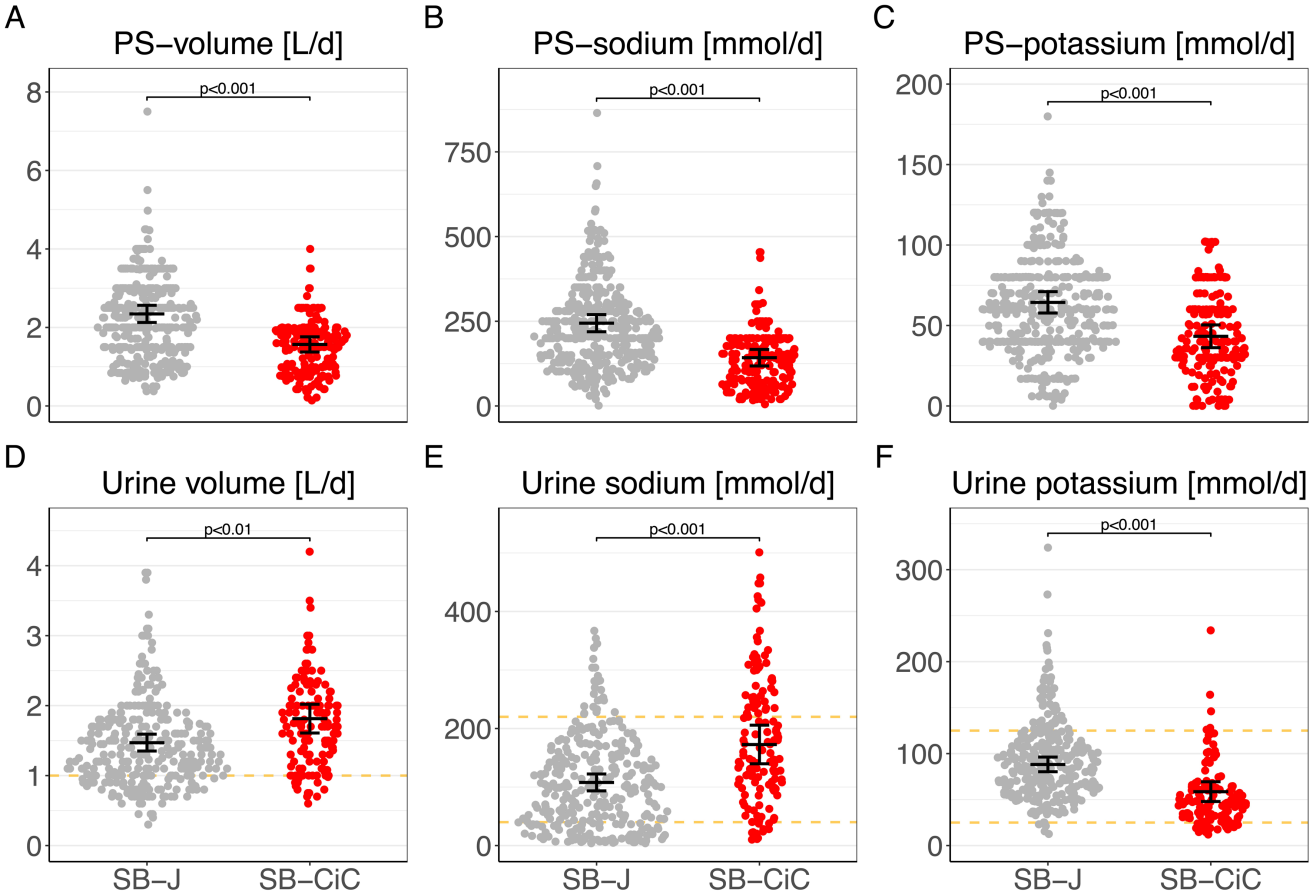
Components of parenteral support **(A–C)** and urine outputs **(D–F)** for SB-J (grey) and SB-CiC (red). Comparisons were calculated with a linear model including patient as a random effect variable to account for repeated measurements. Error bars indicate 95% CI of the estimated mean. Dashed lines indicate reference range. SB-J, short bowel with end-jejunostomy; SB-CiC, short bowel with colon in continuity.

Figures 2A–C shows the daily infusion of parenteral volume, sodium, and potassium for the two anatomical groups. At the cohort level, the mean PS-volume was 1.97 ± 0.97 L/d, the mean PS-sodium was 203 ± 123 mmol/d, and the mean PS-potassium was 55 ± 30 mmol/d. SB-J received significantly higher amounts of all three PS-components compared to SB-CiC. Although patients with SB-J received more volume and sodium, they displayed significantly lower urine volume and urinary sodium excretion than SB-CiC (Figures 2D,E). 20% of measured 24h urine samples from SB-J had a volume of less than 1 L/d compared to 9% in SB-CiC. While only 8% of samples from SB-CiC were below the reference range for 24h sodium excretion, 26% of samples from patients with SB-J were below this reference. These data are consistent with a higher intestinal volume and sodium loss in SB-J. In sharp contrast to volume and sodium, daily urinary potassium excretion was 30 mmol/d (17 to 42; *p* < 0.0001) higher in SB-J than in SB-CiC (Figure 2F).

### 3.3 SB-J is associated with higher aldosterone levels than SB-CiC

**Figure 3:**
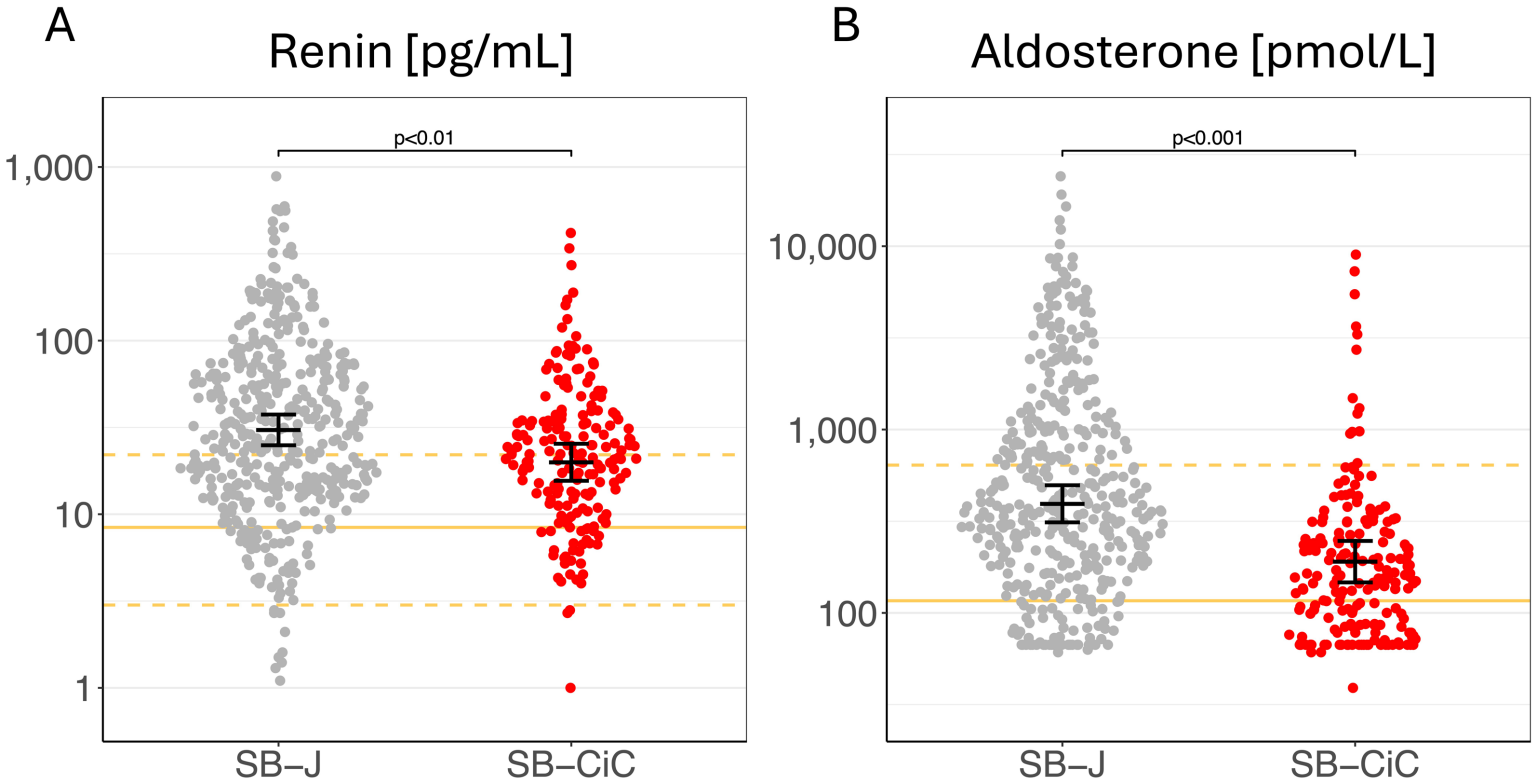
Distribution of renin **(A)** and aldosterone **(B)** in SB-J compared to SB-CiC. Dashed lines indicate reference range. The solid, golden lines indicate median of control cohorts. Each dot represents one measurement. Means and group differences were estimated using a linear model with log10-transformation of the dependent variable and adjustment for multiple observations per patient. Error bars indicate 95% CI of the estimated mean (note log10 scale). SB-J, short bowel with end-jejunostomy; SB-CiC, short bowel with colon in continuity.

Plasma levels of renin and aldosterone are shown in Figure 3. In the total cohort, renin levels were above the upper reference range in 56% of cases, while aldosterone levels were above the upper reference range in 27% of cases. In SB-J, median renin was 1.5-fold higher (CI 1.14 to 2.1; *p* < 0.01) compared to CiC. Congruently, patients with SB-J had 2.1-fold higher aldosterone levels (CI 1.5 to 2.9; *p* < 0.001) and 17.8 times higher odds of hyperaldosteronism (serum level >634 pmol/L) compared to CiC (CI 6.8 to 46.9; *p* < 0.001).

After adjustment for low urinary sodium (urinary sodium excretion <40 mmol/d or urinary sodium concentration <20 mmol/L), aldosterone but not renin levels were significantly higher in SB-J compared to SB-CiC (aldosterone: 1.7-fold, CI 1.2 to 2.4, *p* < 0.01; renin: 1.2-fold, CI 0.85 to 1.7, *p* = 0.31). These estimations indicate that the higher renin levels in SB-J are caused by a greater degree of sodium and/or volume depletion compared to SB-CiC. In contrast, the higher aldosterone levels in SB-J than in SB-CiC appear to be driven by additional factors as well.

### 3.4 Clinical and biochemical predictors of hyperaldosteronism

To determine the mechanisms that cause aldosterone levels to rise above normal values in the clinical scenario of SB requiring PS, we adjusted for multiple parameters using mixed-effect regression models. These models included all observations for which 24h urine collections were available (455 observations). Table 2 shows the odds ratio for hyperaldosteronism and the associated change of serum aldosterone (fold change) for anatomy types and potentially contributing clinical and biochemical parameters. The estimates result from a model including all parameters shown in Table 2 as fixed effect variables. Clinically relevant cut-offs for all continuous parameters were chosen, except for urinary potassium excretion, which was included as a continuous variable due to unclear clinical cut-offs for low, normal, and high urine potassium excretion. Since several of the parameters were collinearly associated, the individual effects of each variable cannot be isolated from this model. If plasma sodium, plasma potassium, urine sodium (sodium excretion <40 mmol/d or sodium concentration <20 mmol/L), and urine volume were integrated as continuous variables, the results did not change substantially, except that the difference between SB-J and SB-CiC was non-significant.

**Table 2:** Association of anatomy types, clinical and biochemical parameters with serum aldosterone.

Parameter	Odds ratio for hyperaldosteronism	Fold higher serum aldosterone
Anatomy type		
SB-J vs. SB-CiC	**5.19 (1.51 to 17.8)**	**1.56 (1.2 to 2.03)**
Plasma renin		
High (>22 pg./mL) vs. normal	**2.87 (1.31 to 6.33)**	**1.36 (1.15 to 1.62)**
eGFR		
<30 vs. >30 ml/min/m^2^	**18.8 (3.45 to 103)**	**1.73 (1.09 to 2.73)**
Plasma sodium		
Low (<136 mmol/L) vs. normal	1.59 (0.58 to 4.35)	**1.38 (1.08 to 1.75)**
Urine sodium		
Low (<40 mmol/d or <20 mmol/L) vs. normal	**28.5 (11.1 to 74)**	**4.05 (3.21 to 5.12)**
Urine volume		
Low (<1 L/d) vs. normal	1.39 (0.53 to 3.62)	1.19 (0.96 to 1.48)
Urine potassium		
Per 10 mmol/d	**1.11 (1.02 to 1.22)**	**1.04 (1.01 to 1.06)**

Estimates were calculated using an adjusted linear mixed-effect model including all variables listed in the first column. In contrast to the models used for the figures, the variables (excluding urine potassium) were dichotomized at clinically relevant cut-offs as also indicated in the first column. 95% CI are reported in parentheses. Significant results are highlighted in bold. SB-J, short bowel with end-jejunostomy; SB-CiC, short bowel with colon in continuity.

Interestingly, if urinary potassium excretion was excluded from the model, the risk of hyperaldosteronism (OR: 8, CI 2.24 to 28) and serum aldosterone levels (1.77-fold higher, CI 1.37 to 2.29) were even higher in SB-J compared to SB-CiC. Therefore, the higher aldosterone levels and the greater risk of hyperaldosteronism could be partly attributed to a higher need for urinary potassium excretion associated with SB-J. These data suggest that, alongside sodium and volume depletion, the need to excrete potassium may be considered an additional factor that contributed to the stimulation of aldosterone secretion in SB-J patients.

### 3.5 Impact of reduced renal function on aldosterone levels

**Figure 4:**
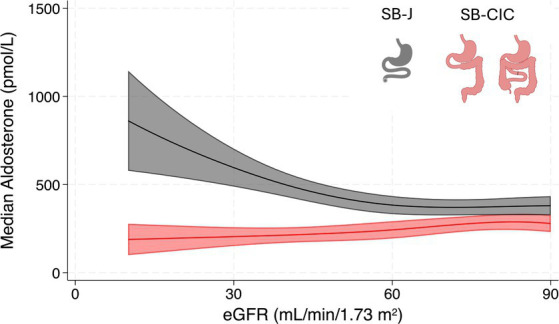
Predicted association of aldosterone with eGFR. This model included anatomy type (SB-J vs. CIC), plasma potassium, plasma sodium, urine volume (low vs. normal), and urinary sodium excretion (low vs. normal) as fixed effects variables. The association between plasma aldosterone and eGFR was significantly affected by anatomical type (*p* = 0.03). Shaded areas show standard error of the predicted mean. SB-J (black), short bowel with end-jejunostomy; SB-CiC (red), short bowel with colon in continuity. Pictogram created in BioRender.com.

The unadjusted odds of hyperaldosteronism were 6.4 times higher (CI 1.04 to 39, *p* < 0.01) for patients with eGFR below 30 mL/min/1.73 m^2^ as compared to patients with eGFR above 30 mL/min/1.73 m^2^. The odds were even higher when adjusting for all the parameters in Table 2 (OR 18.8, CI 3.45 to 103, *p* = 0.001). No association between eGFR and aldosterone levels was found using eGFR as a linear variable. However, allowing for a non-linear relationship between eGFR and aldosterone, an increase in aldosterone levels was observed in SB-J when eGFR was below 45 mL/min/1.73 m^2^ (Figure 4). In patients with SB-J, predicted renin also increased when eGFR decreased. However, due to large standard errors, no clear difference was observed compared to SB-CiC (not shown). These data indicate that a low GFR independently drives increased plasma aldosterone, primarily in patients with SB-J.

### 3.6 Functional anatomy influences the association of aldosterone and renin with physiological predictors

**Figure 5:**
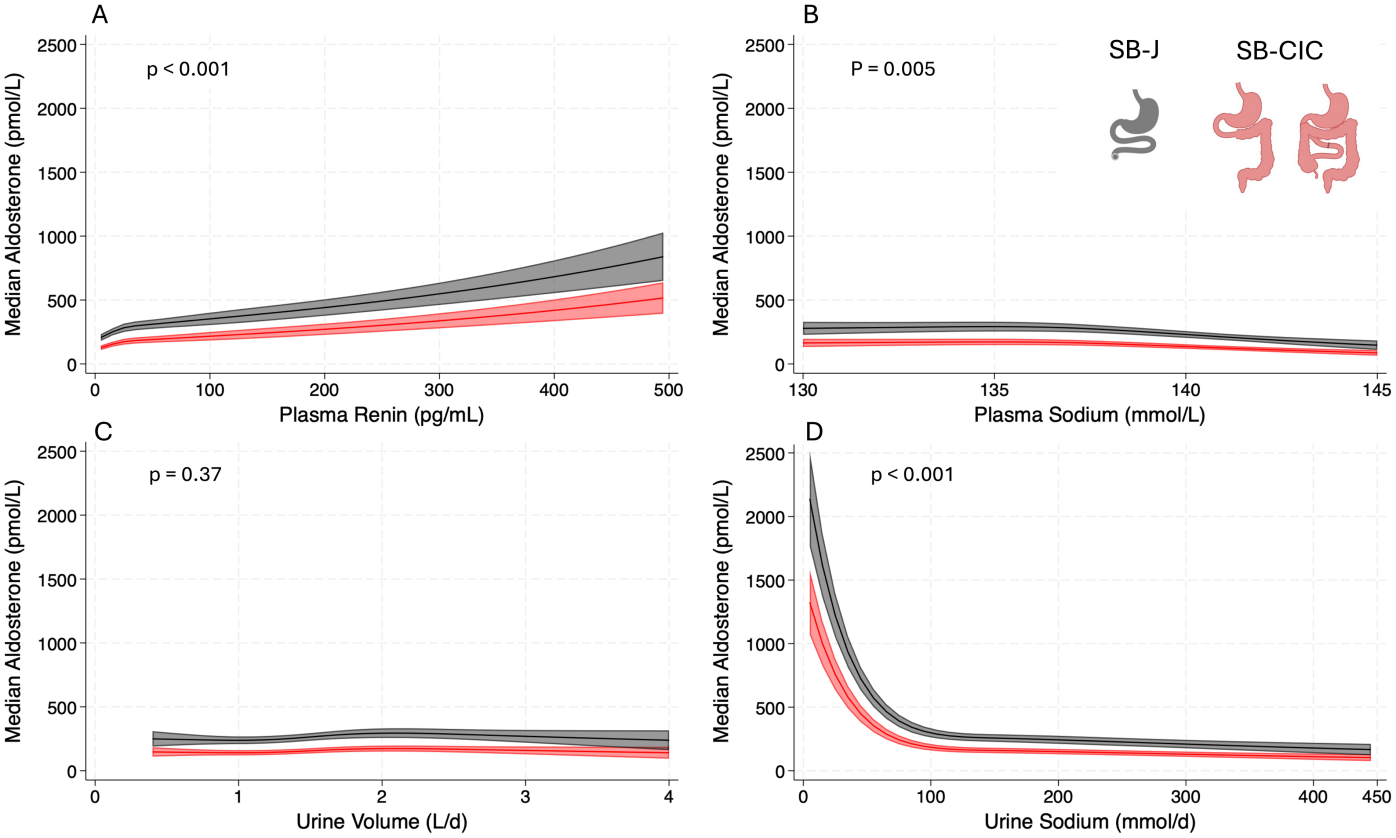
Predicted relationship of median aldosterone with plasma renin **(A)**, plasma sodium **(B)**, urine volume **(C)**, and urine sodium excretion **(D)**. The *p*-values in the figure indicate whether the parameter on the x-axis is associated with aldosterone levels. The model included anatomy type (SB-J vs. CIC), eGFR, plasma sodium, urine volume, and urinary sodium excretion as fixed effect variables. A significant difference in aldosterone levels between anatomical types (*p* < 0.001) was observed. Renin was only added to the model as a fixed effect variable for **(A)**. Shaded areas show standard error of the geometric mean. SB-J (black), short bowel with end-jejunostomy; SB-CiC (red), short bowel with colon in continuity. Pictogram created in BioRender.com.

To better understand how the SB anatomical type affects aldosterone levels, we modeled the relationships between aldosterone and the following markers of volume and electrolyte homeostasis: plasma renin, plasma sodium, urine volume, and urinary sodium excretion. Adjustments for eGFR as a fixed effect were included. Compared to SB-CiC, higher aldosterone levels were detected in SB-J at any given plasma renin, plasma sodium, urine volume, and urinary sodium excretion (Figure 5). All these parameters, except urine volume, were significantly associated with aldosterone. Aldosterone levels were higher in both female and male patients with SB-J in sex-stratified analyses. Interestingly, in the model where variables were included as continuous variables, females appeared to have higher plasma aldosterone levels (1.36-fold higher, CI 1.02 to 1.82, *p* = 0.042) and a higher risk of hyperaldosteronism (3.2 times higher risk, CI 1.2 to 8.7, *p* = 0.02) than males. We observed the strongest exponential association between aldosterone and low urinary sodium excretion (Figure 5D). Interestingly, low urine volume was only associated with higher aldosterone levels when urinary sodium excretion was excluded from the model. As such, urine volume does not seem to provide additional information on volume homeostasis beyond what is given by urinary sodium excretion.

**Figure 6:**
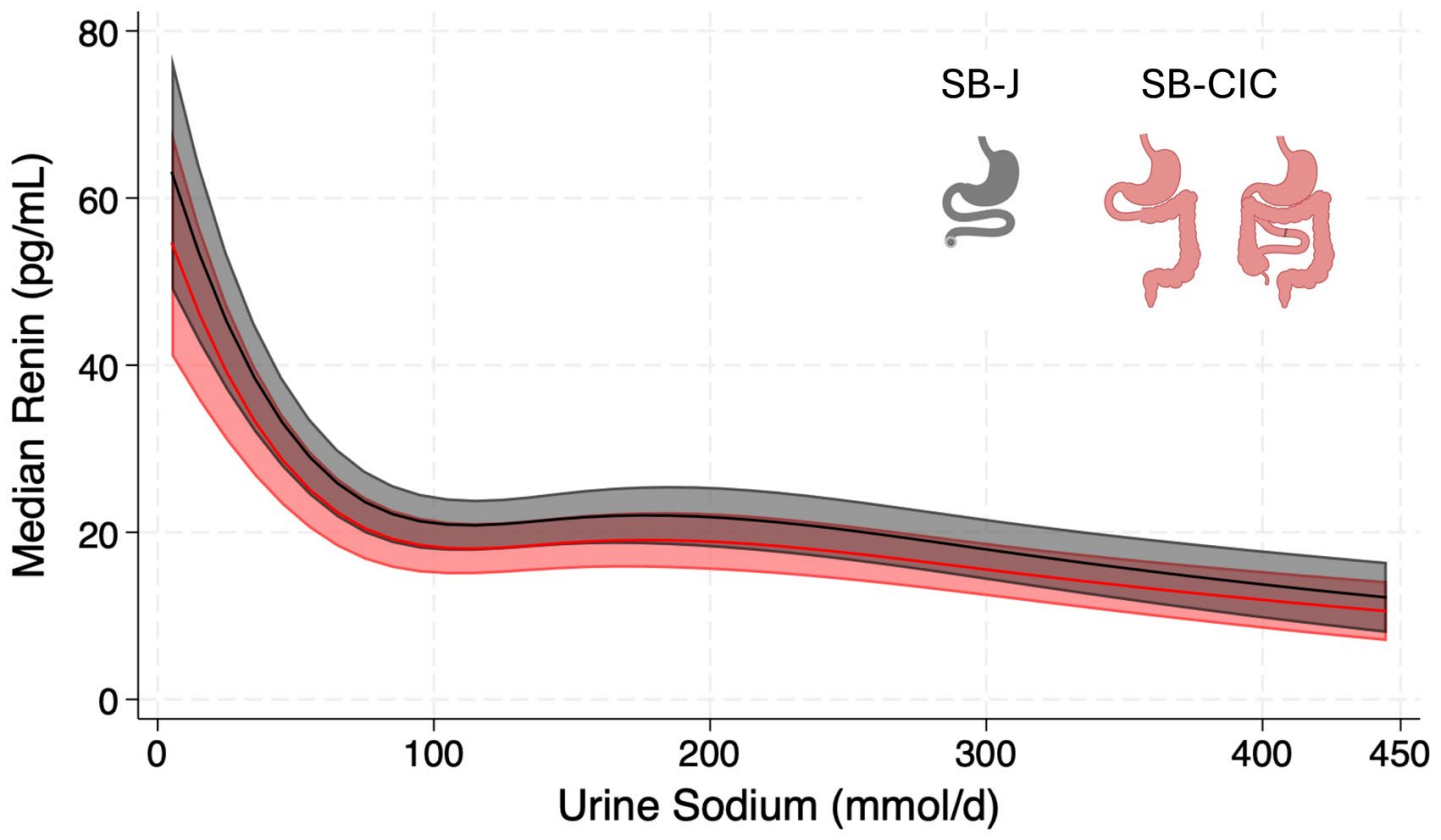
Prediction of median renin over urine sodium excretion. The model included anatomy type (SB-J vs. CIC), eGFR, plasma sodium, urine volume, and urinary sodium excretion as fixed effect variables. A significant association between renin and urinary sodium excretion (*p* < 0.001) was observed. But there was no difference between anatomical types (*p* = 0.36). Shaded areas show standard error of the geometric mean. SB-J (black): short bowel with end-jejunostomy; SB-CiC (red): short bowel with colon in continuity. Pictogram created in BioRender.com.

Since renin precedes aldosterone production via the RAAS, we investigated whether renin levels were also related to the anatomical type of SB (Figure 6). Like plasma aldosterone levels, predicted median renin levels (adjusted for volume and sodium depletion markers in the same manner) also showed exponential elevation as urinary sodium excretion decreased. However, unlike aldosterone levels (Figure 5D), we observed no difference in renin levels between anatomical types (Figure 6). This finding suggests that the renin-dependent activation of the RAAS responds identically to sodium and volume depletion across anatomical SB types.

### 3.7 Higher need for potassium excretion contributes to higher aldosterone in SB-J

**Figure 7:**
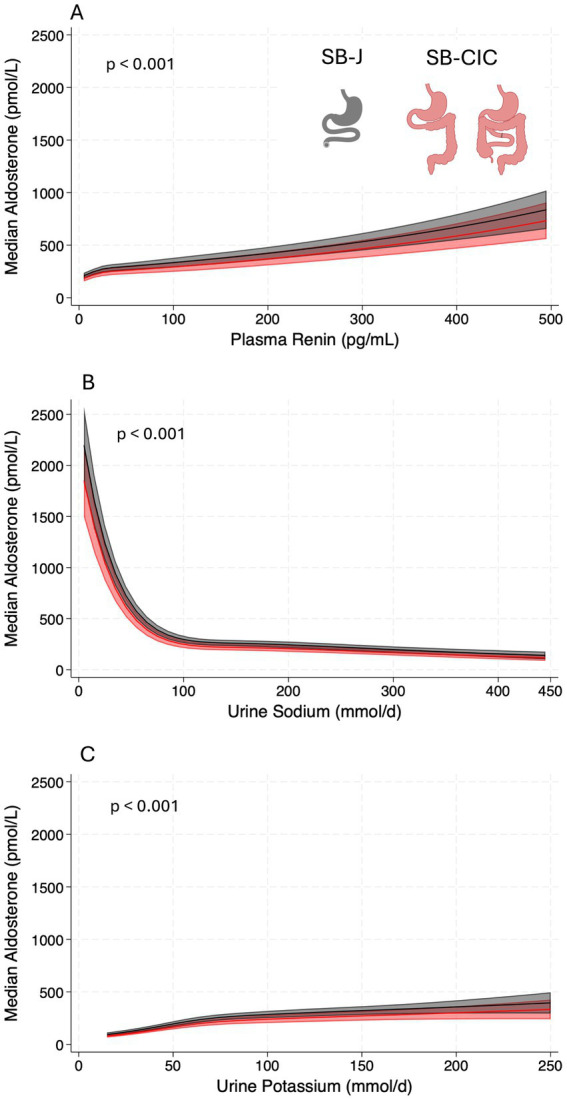
Predicted relationship between median aldosterone and plasma renin **(A)**, urinary sodium excretion **(B)**, and urinary potassium excretion **(C)** in a model which, in contrast to Figure 5, additionally includes urinary potassium excretion as fixed effect. Plasma renin was only included in panel **(A)** as fixed effect (as in Figure 4). The *p*-values in the figure signify whether the parameter on the x-axis is associated with aldosterone levels. There was no difference between anatomical types (**A**: *p* = 0.29; **B**,**C**: *p* = 0.20). Shaded areas show standard error of the median. SB-J (black), short bowel with end-jejunostomy; SB-CiC (red), short bowel with colon in continuity. Pictogram created in BioRender.com.

When we extended the model used for Figure 5 by additionally adjusting for urinary potassium excretion, the robust association between serum aldosterone and both plasma renin and urinary sodium excretion remained significant (Figure 7). Strikingly, no differences in aldosterone levels were found between SB-J and SB-CiC (with renin (Figure 7A): 1.14-fold, CI 0.89 to 1.46, *p* = 0.29; without renin (Figures 7B,C): 1.19-fold, CI 0.91 to 1.54, *p* = 0.20), as illustrated by the overlapping curves. These results support the conclusion that the difference in aldosterone levels between SB-J and SB-CiC, which persists when adjusting for renin-induced volume and sodium regulation (see Figure 5), is caused by differences in the need for potassium excretion.

Congruently, a higher need for potassium excretion and higher levels of aldosterone in SB-J compared to SB-CiC were found in two specific subgroups. Subgroup A included 83 patients (306 observations) who did not have sodium or volume depletion. This was defined as having a urine volume >1.0 L/d and a urinary sodium excretion >40 mmol/d (+35 mmol/d urinary potassium excretion, CI 18 to 52, *p* < 0.001). Subgroup B contained 101 patients (451 observations) without hyperaldosteronism, which was defined as having plasma aldosterone <634 pmol/L (+28 mmol/d urinary potassium excretion, CI 13 to 42, *p* < 0.001). In both subgroups, aldosterone levels were significantly higher in SB-J (subgroup A: 1.7-fold, CI 1.3 to 2.3, *p* < 0.001; subgroup B: 1.3-fold, CI 1.09 to 1.6, *p* < 0.01). These data further support the conclusion that patients with SB-J have a higher need for urinary potassium excretion independent of sodium and volume homeostasis.

### 3.8 Potassium excretion is higher in SB-J even with low potassium infusion

As shown in Figure 2, the urinary excretion of volume (water), sodium, and potassium, as well as PS differed between anatomical types. Therefore, the effects of PS as a specific intervention for IF need to be considered.

**Figure 8:**
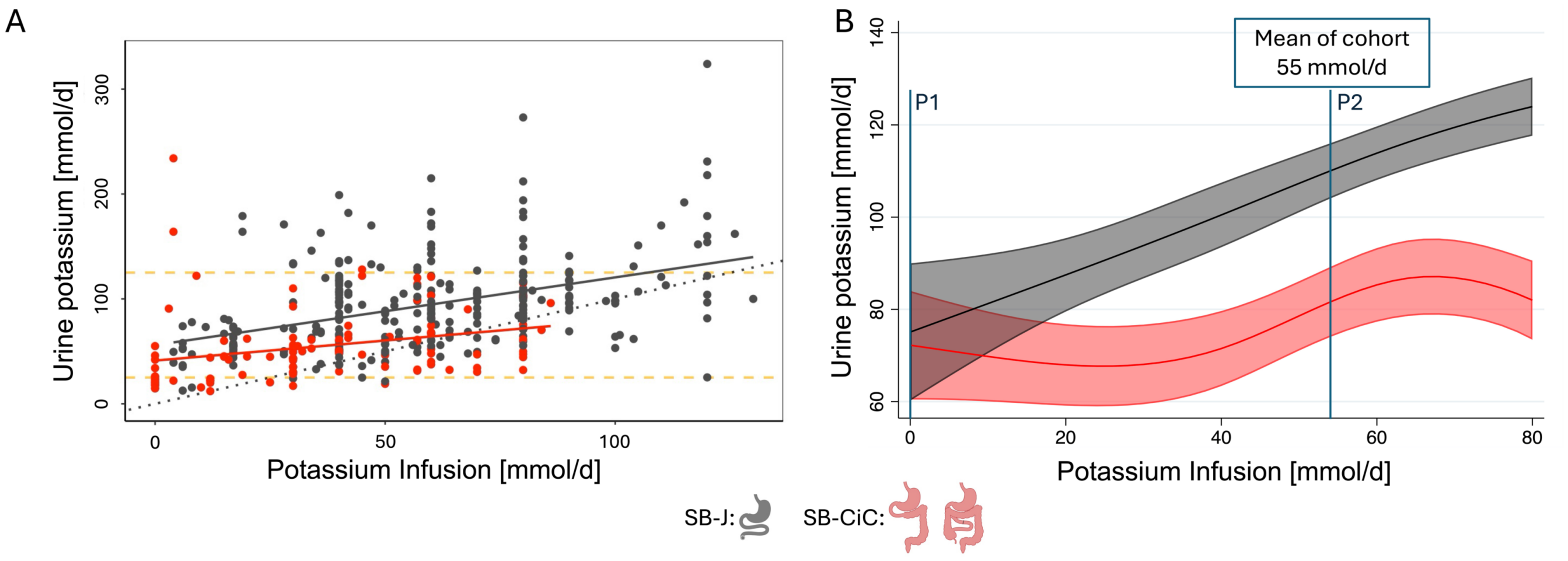
**(A)** Dotplot of urine potassium excretion by potassium infusion per day. Each dot represents one measurement of a patient with small bowel length >50 cm. The black dotted line represents the equilibrium line where potassium infusion equals urinary potassium excretion. Dashed, golden lines indicate reference range. **(B)** Urinary potassium excretion estimated by a model including anatomy type (SB-J vs. SB-CiC), eGFR, plasma potassium, plasma sodium, urinary sodium excretion, sodium infusion, and potassium infusion (allowing a non-linear association) as fixed effect variables. Predictions at potassium infusion = 0 mmol/d (P1) showed no difference. However, at the mean potassium infusion of 55 mmol/d (P2), urinary potassium excretion was significantly higher in SB J (+28 (9 to 47), *p* < 0.01). Anatomical type significantly affected the association between potassium infusion and urinary potassium excretion (*p* = 0.05). Shaded areas show standard error of the predicted mean. SB-J (black): short bowel with end-jejunostomy; SB-CiC (red): short bowel with colon in continuity. Pictogram created in BioRender.com.

Figure 8A shows that, in patients with SB-J, urinary potassium excretion was higher than the amount of parenteral potassium infused at each level of infusion. However, this regression was less steep in patients with SB-CiC. Here, high rates of parenteral potassium infusion were associated with lower urinary potassium excretion than infusion. Since daily urinary potassium excretion was significantly higher in SB-J compared to SB-CiC (Figure 2F), we adjusted for eGFR, serum sodium, serum potassium, sodium excretion, sodium infusion, potassium infusion, and aldosterone as potential covariates. After this adjustment, urinary potassium excretion was 28 mmol/d (CI 9 to 47; *p* < 0.01) higher in SB-J compared to SB-CiC (Figure 8B, P2). This finding was consistent in both males and females in sex-stratified analyses. If potassium excretion was predicted at an assumed potassium infusion of zero (instead of the mean of the cohort, which was 55 mmol/d), there was no significant difference between anatomical types (Figure 8B, P1). Figure 8B shows that this model predicted different slopes for the association between potassium excretion and potassium infusion for the two anatomical types.

The same conclusions were drawn when examining body weight-adjusted potassium infusions instead of the absolute amount of potassium in the PS. In our cohort, the mean potassium infusion was 0.88 mmol/kg/d and this model yielded a 33.7 mmol/d (CI 20.2 to 47.1; *p* < 0.001) higher urinary potassium excretion in SB-J compared to SB-CiC.

These data suggest that urinary potassium excretion in SB-J is linearly stimulated by potassium infusion, beginning to increase even when only a small amount of potassium is provided in the PS. On the other hand, in SB-CiC, urinary potassium excretion is only stimulated in response to a PS potassium load of more than 40 mmol/d.

### 3.9 Prediction of elevated plasma aldosterone levels by urinary electrolyte pattern

Table 3 shows sensitivity, specificity, and the predictive value of urine electrolyte patterns to predict plasma aldosterone levels >634 pmol/L. A urinary sodium-to-potassium ratio of <1 had the highest sensitivity (85%), and adding low urinary sodium concentration (<20 mmol/L) as an optional predictor did not improve this result. Consequently, the absence of both a urinary sodium-to-potassium ratio of <1 and a low urinary sodium concentration (<20 mmol/L) ruled out elevated aldosterone levels, with a negative predictive value of 93%. If urinary sodium concentration was <20 mmol/L, the specificity (98%) and positive predictive value (88%) were most accurate. In the sensitivity analysis evaluating diagnostic accuracy in subgroups excluding patients with diuretics or eGFR <30 mL/min/1.73m^2^, performance was very similar, with slightly different results in line with the different prevalence of elevated plasma aldosterone among the subgroups (Supplementary Table 11).

**Table 3:** Diagnostic accuracy of urine electrolyte concentrations in predicting elevated plasma aldosterone levels.

Urine electrolyte pattern	True positives *n*	False positives *n*	False negatives *n*	True negatives *n*	Sensitivity	Specificity	Positive predictive value	Negative predictive value
U-Na/K-ratio <1	142	97	25	354	**85%**	79%	60%	**93%**
U-Na/K-ratio <1 or U-Na < 20 mmol/L	142	97	25	354	**85%**	79%	60%	**93%**
U-Na < 20 mmol/L	83	11	84	440	50%	**98%**	**88%**	84%

Elevated plasma aldosterone levels were defined as greater than 634 pmol/L, which is the upper limi**t** of normal. Urine electrolyte concentrations were measured in spot urine or 24h urine. The highest value in each column was highlighted in bold. U-Na/K-ratio: urinary sodium-to-potassium ratio. U-Na, urinary sodium concentration.

## 4 Discussion

Parenteral support in intestinal failure aims to restore and maintain water and electrolyte homeostasis. However, both the disease and suboptimal parenteral support trigger counterregulatory responses, particularly changes in renal electrolyte and water excretion. We hypothesized that physiological aldosterone levels indicate minimal counter-regulation and could help establish physiologically optimal parenteral sodium and potassium supplementation.

### 4.1 Key findings

Among all anatomical types, hyperaldosteronism was present in 27% of measurements. The finding of an exponential relationship between low urinary sodium excretion and elevated aldosterone levels confirms the role of aldosterone as the major regulator of sodium and volume homeostasis. Patients with SB-J demonstrated a higher prevalence of volume and sodium depletion. Compared with SB-CiC, SB-J was associated with aldosterone levels that were 2.1 times higher and had an odds ratio of 18 for hyperaldosteronism. Thus, the need to increase parenteral sodium supplementation was more common in SB-J than in SB-CiC, despite that most of the patients received more parenteral sodium than broadly recommended by the recent ESPEN practical guideline (5).

Strikingly, our modeled data revealed that higher aldosterone levels were associated with a greater potassium excretion, likely due to a higher need for potassium excretion following overzealous intravenous potassium supplementation. This occurred independently of any disturbances in volume and sodium homeostasis (21, 22). Of note, patients with SB-J had a significantly greater requirement for urinary potassium excretion than patients with SB-CiC, even after adjusting for the different amounts of intravenous potassium supplementation. Thus, in our patients, parenteral potassium supplementation often overcompensated for the estimated intestinal potassium losses attributed to small intestinal stoma output or colonic diarrhea. Remarkably, this occurred even though most of our patients received less parenteral potassium than recommended by the recent ESPEN practical guideline (5).

### 4.2 Factors driving high plasma aldosterone in intestinal failure

Regarding potassium supplementation, two physiological mechanisms may not have been sufficiently recognized previously. Firstly, even under IF conditions, the small intestine net absorbs potassium taken up orally when its length is at least 50 cm (5, 23, 24). A large proportion (90%) of our patients with SB-J have more than 50 cm of remaining small bowel. Based on another cohort of 28 orally compensated patients with SB at our center, we estimate dietary potassium intake of 91 ± 36 mmol/d (25). Thus, dietary potassium was substantially absorbed in the residual proximal small bowel, which reduced the actual need for parenteral potassium support.

Secondly, if present, the colon maintains regulated potassium secretion in response to chronic potassium loading (26–28). Under physiological conditions, the range of colonic potassium loss has been reported between 2 and 20 mmol/d, which can increase to 55 mmol/d or more in pathophysiological states like ‘colonic’ diarrhea (29, 30). In our study, patients with SB-J compensate for the lack of colonic excretion with additional urinary excretion of 28 mmol of potassium per day, which is induced by higher aldosterone activation.

Taken together, our modeled analysis confirms that, in intestinal failure, aldosterone is strongly triggered by sodium depletion and, to a lesser extent, by the need for potassium excretion. The latter usually does not reach hyperaldosteronism thresholds and thus may go undetected if only pathologically elevated aldosterone values are considered.

### 4.3 Clinical implications for optimal parenteral sodium and potassium support

Our analysis provides quantitative data on the diagnostic performance of urine electrolytes for predicting hyperaldosteronism in IF. As a result, targeting a urinary sodium concentration of >20 mmol/L and a urinary sodium-to-potassium ratio of >1 is a simple way to ensure optimal provision of parenteral sodium and potassium with a high degree of reliability in IF patients (negative predictive value for hyperaldosteronism 93%). Therefore, the benefit of using aldosterone as an indicator for suboptimal PS in addition to urine electrolytes seems limited to specific clinical considerations, such as renal tubular sodium loss. Operationally, water and sodium supplementation should primarily be optimized based on an adequate urine volume and urinary sodium excretion >20 mmol/L. Second, the amount of potassium supplementation should be chosen based on the estimated net absorptive capacity of the remaining small intestine, plus the ‘tonic’ secretory potassium loss from the colon, in the case of SB-CiC. Based on our physiological data, potassium supplementation of 1.0 mmol/kg/d or less appears to be sufficient for most patients with intestinal failure, especially those with SB-J. This recommendation differs from the current ESPEN recommendation and needs prospective validation. No clear cut-off values for urine potassium excretion have been established. In clinical practice, the reduction of PS potassium can be monitored by tracking urinary potassium excretion over time. Our data suggest that, once sodium and water homeostasis are maintained, PS potassium should be titrated to achieve a urine sodium/potassium ratio of >1. In practice, this may require PS-compounding instead of using standard bags.

### 4.4 Strengths of mixed-effects models using real-world longitudinal data

The important strength of this study is the use of repeated routine monitoring data obtained during long-term follow-up. The dataset comprised 618 measurements from 110 patients, with a median of three observations per patient, reflecting routine quarterly clinical monitoring.

Such real-world, longitudinal data offer several advantages for studying electrolyte physiology in intestinal failure. First, repeated measurements capture dynamic changes in electrolyte balance and treatment adjustments over time, which cannot be adequately assessed in cross-sectional analyses. Although these treatment adjustments inevitably influence the observed parameters, they also provide valuable information about the physiological responses of the RAAS to varying sodium and potassium loads. Importantly, the mixed-effects models used in this study allow for the separation of within-patient and between-patient variability by accounting for patient-specific baseline differences through random effects. The sensitivity analyses performed on subgroups that excluded patients with an eGFR <30 mL/min/1.73m^2^, any imputed data, or missing 24h urine collection, respectively, supported the robustness of the findings (Supplementary Tables 2–10).

### 4.5 Limitations

Limitations of our findings primarily stem from the observational design of the study, which limits causal inference. However, prospective studies addressing electrolyte homeostasis in IF are rarely feasible in large cohorts of clinically stable patients. Second, renin and aldosterone levels were obtained during clinical sampling without standardized resting periods, which may have influenced absolute hormone concentrations. Nevertheless, this limitation is unlikely to affect the observed associations between aldosterone levels and urinary electrolyte parameters. Further, certain patient subgroups may be underrepresented. For example, the low prevalence of eGFR <30 mL/min/1.73m^2^ in our cohort reduces the accuracy of the results for this specific group. Furthermore, oral intake and stoma output were not systematically recorded, preventing a complete analysis of electrolyte balance. Finally, the accuracy of urine parameters to predict hyperaldosteronism depends on the prevalence of hyperaldosteronism in the cohort under consideration. Therefore, to formally establish definitive clinical standards, it is necessary to validate the proposed urinary cut-offs externally, while taking into account potential variations in clinical characteristics, such as the renal function within the examined cohort.

## 5 Conclusion

In patients with intestinal failure, elevated aldosterone levels are primarily driven by sodium depletion and, to a lesser extent, by increased demand for potassium excretion. The latter appears particularly relevant in patients with SB-J who lack physiologically regulated colonic potassium secretion. Routine urine electrolyte monitoring provides reliable information about sodium balance and RAAS activation. Using the physiological regulator aldosterone in a large clinical cohort as a biomarker and applying model analysis, we show that a urine sodium concentration >20 mmol/L combined with a urinary sodium-to-potassium ratio >1 can rule out stimulated aldosterone secretion in IF with a high degree of certainty. Thus, these target values indicate physiologically optimal sodium and potassium support. For most patients with IF, parenteral potassium support of 1.0 mmol/kg/d or less appears sufficient. In addition to the existing algorithmic optimization of volume support these target values will help to establish optimal sodium and potassium support in patients with IF.

## Data Availability

The raw data supporting the conclusions of this article will be made available by the authors, without undue reservation.

## References

[1] Pironi L Arends J Baxter J Bozzetti F Peláez RB Cuerda C . ESPEN endorsed recommendations. Definition and classification of intestinal failure in adults. Clin Nutr. (2015) 34:171–80. doi: 10.1016/j.clnu.2014.08.017, 25311444

[2] Pironi L. Hébuterne X. Van Gossum A. Messing B. Lyszkowska M. Colomb V. Candidates for intestinal transplantation: a multicenter survey in Europe. Am J Gastroenterol (2006) 101:1633–1643. doi:doi: 10.1111/j.1572-0241.2006.00710.x 16863571

[3] Verbiest A Jeppesen PB Joly F Vanuytsel T. The role of a Colon-in-continuity in short bowel syndrome. Nutrients. (2023) 15:628. doi: 10.3390/nu15030628, 36771335 PMC9918966

[4] Nightingale JMD. How to manage a high-output stoma. Frontline Gastroenterol. (2022) 13:140–51. doi: 10.1136/flgastro-2018-101108, 35300464 PMC8862462

[5] Cuerda C Pironi L Arends J Bozzetti F Gillanders L Jeppesen PB . ESPEN practical guideline: Clinical nutrition in chronic intestinal failure. Clin Nutr. (2021) 40:5196–220. doi: 10.1016/j.clnu.2021.07.002, 34479179

[6] Jeppesen PB Vanuytsel T Subramanian S Joly F Wanten G Lamprecht G . Glepaglutide, a long-acting glucagon-like Peptide-2 analogue, reduces parenteral support in patients with short bowel syndrome: a phase 3 randomized controlled trial. Gastroenterology. (2025) 168:701–713.e6. doi: 10.1053/j.gastro.2024.11.023, 39708985

[7] Jeppesen PB Pertkiewicz M Messing B Iyer K Seidner DL O’keefe SJD . Teduglutide reduces need for parenteral support among patients with short bowel syndrome with intestinal failure. Gastroenterology. (2012) 143:1473–1481.e3. doi: 10.1053/j.gastro.2012.09.007 22982184

[8] Nightingale JMD. Clinical problems of a short bowel and their treatment. Proc Nutr Soc. (1994) 53:373–91. doi: 10.1079/PNS19940043, 7972152

[9] Debongnie JC Phillips SF. Capacity of the human colon to absorb fluid. Gastroenterology. (1978) 74:698–703. doi: 10.1016/0016-5085(78)90246-9, 631507

[10] Lauverjat M Hadjaissa A Vanhems P Bouletreau P Fouque D Chambrier C. Chronic dehydration may impair renal function in patients with chronic intestinal failure on long-term parenteral nutrition. Clin Nutr. (2006) 25:75–81. doi: 10.1016/j.clnu.2005.09.010, 16356596

[11] Fountain JH Kaur J Lappin SL. Physiology, Renin Angiotensin System. Treasure Island (FL): StatPearls Publishing (2025). 29261862

[12] Bartter FC Liddle GW Duncan LE Barber JK Delea C. The regulation of aldosterone secretion in man: the role of fluid volume. J Clin Invest. (1956) 35:1306–15. doi: 10.1172/JCI103386, 13376724 PMC441709

[13] Sørensen MV Saha B Jensen IS Wu P Ayasse N Gleason CE . Potassium acts through mTOR to regulate its own secretion. JCI Insight. (2019) 5:e126910. doi: 10.1172/jci.insight.126910 31013253 PMC6629116

[14] Willenberg HS Schinner S Ansurudeen I. New mechanisms to control aldosterone synthesis. Horm Metab Res. (2008) 40:435–41. doi: 10.1055/s-2008-1065336 18493881

[15] Hattangady N Olala L Bollag WB Rainey WE. Acute and chronic regulation of aldosterone production. Mol Cell Endocrinol. (2012) 350:151–62. doi: 10.1016/j.mce.2011.07.034, 21839803 PMC3253327

[16] Pironi L Arends J Bozzetti F Cuerda C Gillanders L Jeppesen PB . ESPEN guidelines on chronic intestinal failure in adults. Clin Nutr. (2016) 35:247–307. doi: 10.1016/j.clnu.2016.01.020, 26944585

[17] Kirby DF Dudrick SJ. Practical Handbook of Nutrition in Clinical Practice.CRC Press (1994). p. 320.

[18] Cummings JH James WPT Wiggins HS. Role of the colon in ileal-resection diarrhea. Lancet. (1973) 301:344–7. doi: 10.1016/S0140-6736(73)90131-1, 4121937

[19] Levey AS Stevens LA Schmid CH Zhang YL Castro AF Feldman HI . A new equation to estimate glomerular filtration rate. Ann Intern Med. (2009) 150:604–12. doi: 10.7326/0003-4819-150-9-200905050-00006 19414839 PMC2763564

[20] Hannemann A Friedrich N Lüdemann J Völzke H Rettig R Peters J . Reference intervals for aldosterone, renin, and the aldosterone-to-renin ratio in the population-based study of health in Pomerania (SHIP-1). Horm Metab Res. (2010) 42:392–9. doi: 10.1055/s-0030-1247545, 20157876

[21] Martin RS Hayslett JP. Role of aldosterone in the mechanism of renal potassium adaptation. Pflüg Arch. (1986) 407:76–81. doi: 10.1007/BF00580724, 3737385

[22] Todkar A Picard N Loffing-Cueni D Sorensen MV Mihailova M Nesterov V . Mechanisms of renal control of potassium homeostasis in complete aldosterone deficiency. J Am Soc Nephrol. (2015) 26:425–38. doi: 10.1681/ASN.2013111156, 25071088 PMC4310654

[23] Nightingale JMD Lennard-Jones JE Walker ER Farthing MJG. Jejunal efflux in short bowel syndrome. Lancet. (1990) 336:765–8. doi: 10.1016/0140-6736(90)93238-K, 1976145

[24] Ladefoged K ØLgaard K. Fluid and electrolyte absorption and renin-angiotensin-aldosterone axis in patients with severe short-bowel syndrome. Scand J Gastroenterol. (1979) 14:729–35. doi: 10.3109/00365527909181945, 119306

[25] Bannert K Karbe C Förster RH Sautter LF Meyer F Valentini L . Orally compensated short bowel patients are thin, potentially malnourished but rarely sarcopenic. Clin Nutr. (2023) 42:1480–90. doi: 10.1016/j.clnu.2023.05.018, 37311685

[26] Dolman D Edmonds CJ. The effect of aldosterone and the renin-angiotensin system on sodium, potassium and chloride transport by proximal and distal rat colon in vivo. J Physiol. (1975) 250:597–611. doi: 10.1113/jphysiol.1975.sp011072, 1177152 PMC1348395

[27] Sweiry JH Binder HJ. Characterization of aldosterone-induced potassium secretion in rat distal colon. J Clin Invest. (1989) 83:844–51. doi: 10.1172/JCI113967, 2921323 PMC303757

[28] Sorensen MV Matos JE Praetorius HA Leipziger J. Colonic potassium handling. Pflug Arch Eur J Physiol. (2010) 459:645–56. doi: 10.1007/s00424-009-0781-9, 20143237

[29] Agarwal R Afzalpurkar R Fordtran JS. Pathophysiology of potassium absorption and secretion by the human intestine. Gastroenterology. (1994) 107:548–71. doi: 10.1016/0016-5085(94)90184-8, 8039632

[30] Blondon H Béchade D Desramé J Algayres J-P. Secretory diarrhoea with high faecal potassium concentrations: a new mechanism of diarrhoea associated with colonic pseudo-obstruction? Report of five patients. Gastroentérologie Clin Biol. (2008) 32:401–4. doi: 10.1016/j.gcb.2007.11.014, 18394839

