# Supplementary material for: Aldosterone-identified targets for optimal sodium and potassium supplementation in intestinal failure
Source: Front Nutr. 2026 Jul 14;13:1864539. doi: 10.3389/fnut.2026.1864539 (PMC13407092; doi:10.3389/fnut.2026.1864539)
Supplement: Supplementary file 1 [file Data_Sheet_1.PDF]

Supplementary file

Concomitant medication

Suppl. Table 1: Concomitant medication during the observation period

Medication	Absolute (Relative)
Diuretics	18 (16 %)
ACE inhibitors	14 (13 %)
Angiotensin II receptor blockers	11 (10 %)
Beta blockers	30 (27 %)
Mineralocorticoid receptor antagonists	3 (3 %)
Systemic corticosteroids	9 (8 %)
Oral supplements	26 (24 %)
Potentially nephrotoxic agents	16 (15 %)

The table summarizes how many patients received the respective medication at any point during the observation period. The group of oral supplements included magnesium, zinc, calcium, potassium, bicarbonate and iron. The group of potentially nephrotoxic agents compromised non-steroidal anti-inflammatory drugs (NSAIDs), ciclosporin A, amphotericin B, bisphosphonates, mesalamine, cyclooxygenase-2 (COX-2) inhibitors, methotrexate and sorafenib.

Sensitivity analysis

Sensitivity analyses were performed for the following subgroups:

1. Observations with eGFR >30 ml/min/1.73m^2 (105 patients; 591 observations)
2. Full case subgroup excluding observations with any imputed data (108 patients; 589 observations)
3. Patients with 24h collection urine only (excluding spot urine samples) (93 patients; 455 observations)

1. Aldosterone and renin in SB-J vs. SB-CiC (Chapter 3.3)

Suppl. Table 2: Comparison of aldosterone and renin between anatomical types with adjustment for low urinary sodium

		**Original cohort**	**Subgroup 1** (GFR >30)	**Subgroup 2** (without imputation)	**Subgroup 3** (24h urine available)
Aldosterone levels	SB-J vs. SB-CiC	1.7-fold, CI 1.2 to 2.4, **p < 0.01**	1.9-fold, CI 1.4 to 2.6, **p < 0.001**	1.6-fold, CI 1.1 to 2.2, **p < 0.05**	1.5-fold, CI 1.1 to 2.2, **p < 0.05**
Renin levels	SB-J vs. SB-CiC	1.2-fold, CI 0.9 to 1.7, p = 0.31	1.3-fold, CI 0.9 to 1.8, p = 0.20	1.2-fold, CI 0.8 to 1.7, p = 0.34	1.2-fold, CI 0.8 to 1.8, p = 0.45

Low urinary sodium was defined as urinary sodium excretion <40 mmol/d or urinary sodium concentration <20 mmol/L.

2. Clinical and biochemical predictors of hyperaldosteronism and high serum aldosterone (Chapter 3.4, Table 2)

Suppl. Table 3: Association of anatomy types, clinical and biochemical parameters with odds ratio for hyperaldosteronism

	Original cohort	Subgroup 1 (GFR >30)	Subgroup 2 (without imputation)
Anatomy type<br>SB-J vs. SB-CiC	**5.19 (1.51 to 17.8)**	3.24 (0.99 to 10.6)	**5.19 (1.51 to 17.9)**
Plasma renin<br>High (>22 pg/mL) vs. normal	**2.87 (1.31 to 6.33)**	**2.66 (1.2 to 5.87)**	**2.88 (1.29 to 6.46)**
eGFR<br><30 vs. >30 ml/min/m^2	**18.8 (3.45 to 103)**	*Not applicable*	**18.6 (3.41 to 102)**
Plasma sodium<br>Low (<136 mmol/L) vs. normal	1.59 (0.58 to 4.35)	1.89 (0.68 to 5.25)	1.60 (0.58 to 4.40)
Urine sodium<br>Low (<40 mmol/d or <20 mmol/L) vs. normal	**28.5 (11.1 to 74)**	**27.3 (10.9 to 68.3)**	**28.0 (10.8 to 72.4)**
Urine volume<br>Low (<1 L/d) vs. normal	1.39 (0.53 to 3.62)	1.30 (0.51 to 3.29)	1.40 (0.53 to 3.64)
Urine potassium<br>Per 10 mmol/d	**1.11 (1.02 to 1.22)**	**1.12 (1.02 to 1.22)**	**1.11 (1.01 to 1.21)**

The applied model adjusts for all parameters listed in the first column. The details of the model are reported in the manuscript (Table 2). The table reports odds ratios for hyperaldosteronism and 95% confidence intervals within brackets.

Suppl. Table 4: Association of anatomy types, clinical and biochemical parameters with serum aldosterone

	Original cohort	Subgroup 1 (GFR >30)	Subgroup 2 (without imputation)
Anatomy type<br>SB-J vs. SB-CiC	**1.56 (1.2 to 2.03)**	**1.55 (1.19 to 2.03)**	**1.54 (1.18 to 2.01)**
Plasma renin<br>High (>22 pg/mL) vs. normal	**1.36 (1.15 to 1.62)**	**1.37 (1.15 to 1.63)**	**1.36 (1.14 to 1.62)**
eGFR<br><30 vs. >30 ml/min/m^2	**1.73 (1.09 to 2.73)**	*Not applicable*	**1.72 (1.08 to 2.74)**
Plasma sodium<br>Low (<136 mmol/L) vs. normal	**1.38 (1.08 to 1.75)**	**1.38 (1.06 to 1.78)**	**1.35 (1.06 to 1.73)**
Urine sodium<br>Low (<40 mmol/d or <20 mmol/L) vs. normal	**4.05 (3.21 to 5.12)**	**4.04 (3.18 to 5.12)**	**4.07 (3.20 to 5.17)**
Urine volume<br>Low (<1 L/d) vs. normal	1.19 (0.96 to 1.48)	1.18 (0.95 to 1.47)	1.21 (0.7 to 1.50)
Urine potassium<br>Per 10 mmol/d	**1.04 (1.01 to 1.06)**	**1.04 (1.01 to 1.06)**	**1.04 (1.01 to 1.06)**

The applied model adjusts for all parameters listed in the first column. The details of the model are reported in the manuscript (Table 2). The table reports fold higher serum aldosterone and 95% confidence intervals within brackets.

Suppl. Table 5: Hyperaldosteronism and aldosterone levels without adjustments for urinary potassium excretion

		Original cohort	Subgroup 1 (GFR >30)	Subgroup 2 (without imputation)
Hyperaldosteronism	SB-J vs. SB-CiC	OR: 8, CI 2.24 to 28	OR: 5.2, CI 1.53 to 17.7	OR: 7.89, CI 2.22 to 28
Aldosterone levels	SB-J vs. SB-CiC	1.77-fold, CI 1.37 to 2.29	1.77-fold, CI 1.36 to 2.29	1.76-fold, CI 1.36 to 2.29

The applied model includes adjustments for all variables listed in Suppl. Table 3 and Suppl. Table 4 excluding urinary potassium excretion.

3. Impact of reduced renal function on aldosterone levels (Chapter 3.5)

Suppl. Table 6: Odds ratio for hyperaldosteronism with reduced eGFR

		**Original cohort**	**Subgroup 2** (without imputation)
unadjusted	eGFR <30 vs. >30 ml/min/1.73 m^2	OR: 6.35, CI 1.04 to 39, **p < 0.01**	OR 7.0, CI 1.09 to 45, **p = 0.040**
adjusted	eGFR <30 vs. >30 ml/min/1.73 m^2	OR 18.8, CI 3.45 to 103, **p = 0.001**	OR 18.6, CI 3.41 to 102, **p = 0.001**

A sensitivity analysis for subgroup 1 is not applicable, as patients with an eGFR <30 ml/min/1.73 m² were excluded by subgroup definition.

4. Effects of functional anatomy, plasma renin, plasma sodium, urine volume, and urine sodium excretion on aldosterone (Chapter 3.6)

Suppl. Table 7: Predicted relationship of median aldosterone with plasma renin, plasma sodium, urine volume, and urine sodium excretion

		**Original cohort**	**Subgroup 1** (GFR >30)	**Subgroup 2** (without imputation)
Plasma renin	Association with aldosterone	**p < 0.001**	**p < 0.001**	**p < 0.001**
Plasma sodium	Association with aldosterone	**p < 0.005**	**p = 0.011**	**p = 0.008**
Urine volume	Association with aldosterone	p = 0.37	p = 0.35	p = 0.42
Urine sodium	Association with aldosterone	**p < 0.001**	**p < 0.001**	**p < 0.001**
	SB-J vs. SB-CiC	**p < 0.001**	**p < 0.001**	**p < 0.001**

The applied model refers to Figure 5 in the manuscript.

Suppl. Table 8: Predicted relationship of median renin with urinary sodium excretion

		**Original cohort**	**Subgroup 1** (GFR >30)	**Subgroup 2** (without imputation)
Urine sodium	Association with renin	**p <0.001**	**p <0.001**	**p <0.001**
	SB-J vs. SB-CiC	p = 0.36	p = 0.34	p = 0.46

The applied model refers to Figure 6 in the manuscript.

5. Adjusted associations with aldosterone in a model including urinary potassium excretion (Chapter 3.7)

Suppl. Table 9: Predicted relationship of median aldosterone with plasma renin, plasma sodium, urine volume, and urine sodium excretion including urinary potassium excretion

		**Original cohort**	**Subgroup 1** (GFR >30)	**Subgroup 2** (without imputation)
Plasma renin	Association with aldosterone	**p < 0.001**	**p < 0.001**	**p < 0.001**
Urine sodium	Association with aldosterone	**p < 0.001**	**p < 0.001**	**p < 0.001**
Urine potassium	Association with aldosterone	**p < 0.001**	**p < 0.001**	**p < 0.001**
	SB-J vs. SB-CiC	With Renin: 1.14-fold, CI 0.89 to 1.46, p = 0.29	With Renin: 1.16-fold, CI 0.91 to 1.49, p = 0.24	With Renin: 1.10-fold, CI 0.85 to 1.42, p = 0.46
		Without Renin: 1.19-fold, CI 0.91 to 1.54, p = 0.20	Without Renin: 1.20-fold, CI 0.92 to 1.56, p = 0.17	Without Renin: 1.13-fold, CI 0.86 to 1.47, p = 0.38

The applied model refers to Figure 7 in the manuscript.

Suppl. Table 10: Urinary potassium excretion estimated by a model including anatomy type (SB-J vs. CIC), eGFR, plasma potassium, plasma sodium, urinary sodium excretion, sodium infusion, and potassium infusion (allowing a non-linear association) as fixed effect variables

		**Original cohort**	**Subgroup 1** (GFR >30)	**Subgroup 2** (without imputation)
Estimated urinary potassium excretion *at 55 mmol/d (mean potassium infusion)*	SB-J vs. SB-CiC	+28 mmol/d, CI 9 to 47, **p <0.01**	+35 mmol/d, CI 22 to 49, **p <0.001**	+36 mmol/d, CI 23 to 49, **p <0.001**
Estimated urinary potassium excretion *at zero potassium infusion*	SB-J vs. SB-CiC	Not significant	Not significant	Not significant
Effect of anatomical type on the association between potassium infusion and urinary potassium excretion		**p = 0.05**	p = 0.06	**p = 0.02**

The applied model refers to Figure 8 in the manuscript. The model includes anatomy type (SB-J vs. SB-CiC), eGFR, plasma potassium, plasma sodium, urinary sodium excretion, sodium infusion, and potassium infusion (allowing a non-linear association) as fixed effect variables.

6. Prediction of elevated plasma aldosterone levels by urinary electrolyte pattern (Chapter 3.9)

Suppl. Table 11: Diagnostic accuracy of urine electrolyte concentrations in predicting elevated plasma aldosterone levels

	True positives *n*	False positives *n*	False negatives *n*	True negatives *n*	Sensitivity	Specificity	Positive predictive value	Negative predictive value
**All observations (n = 618, prevalence of hyperaldosteronism 27%)**								
U-Na/K-ratio <1	142	97	25	354	**85%**	79%	60%	**93%**
U-Na/K-ratio <1 or U-Na <20 mmol/L	142	97	25	354	**85%**	79%	60%	**93%**
U-Na <20 mmol/L	83	11	84	440	50%	**98%**	**88%**	84%
**Observations from spot urine only (n = 159, prevalence of hyperaldosteronism 33%)**								
U-Na/K-ratio <1	49	34	4	72	**92%**	68%	59%	**95%**
U-Na/K-ratio <1 or U-Na <20 mmol/L	49	34	4	72	**92%**	68%	59%	**95%**
U-Na <20 mmol/L	35	4	18	102	66%	**96%**	**90%**	85%
**Observations from 24h urine only (n = 454, prevalence of hyperaldosteronism 25%)**								
U-Na/K-ratio <1	93	63	21	277	**82%**	81%	60%	**93%**
U-Na/K-ratio <1 or U-Na <20 mmol/L	93	63	21	277	**82%**	81%	60%	**93%**
U-Na <20 mmol/L	48	7	66	333	42%	**98%**	**87%**	83%
**Observations from patients without diuretics only (n = 537, prevalence of hyperaldosteronism 28%)**								
U-Na/K-ratio <1	128	74	22	313	**85%**	81%	63%	**93%**
U-Na/K-ratio <1 or U-Na <20 mmol/L	128	74	22	313	**85%**	81%	63%	**93%**
U-Na <20 mmol/L	76	9	74	378	51%	**98%**	**89%**	84%
**Observations from patients with GFR >30 ml/min/m^2 only (n = 591, prevalence of hyperaldosteronism 26 %)**								
U-Na/K-ratio <1	136	96	17	342	**89%**	78%	59%	**95%**
U-Na/K-ratio <1 or U-Na <20 mmol/L	136	96	17	342	**89%**	78%	59%	**95%**
U-Na <20 mmol/L	82	11	71	427	54%	**97%**	**88%**	86%
